# Preliminary psychometric properties of the Brazilian Portuguese version of the emotional outburst questionnaire

**DOI:** 10.1038/s41598-023-49834-3

**Published:** 2024-01-10

**Authors:** Maria Cristina Triguero Veloz Teixeira, Tally Lichtensztejn Tafla, Rosane Lowenthal, Cristiane Silvestre Paula, Bianca Balbueno, Carmel Mevorach, Justin Cheuk Yin Chung, Kate Anne Woodcock

**Affiliations:** 1 Center for Research on Childhood and Adolescence, Human Developmental Sciences Graduate Program at Mackenzie Presbyterian University, São Paulo, SP Brazil; 2 Department of Mental Health, Santa Casa de São Paulo School of Medical Sciences, São Paulo, SP Brazil; 3 Centre of Human Brain Health, School of Psychology, University of Birmingham, Birmingham, UK; 4 School of Psychology, University of Birmingham, Birmingham, UK; 5 Present Address: Centre for Applied Psychology, School of Psychology & Institute for Mental Health, University of Birmingham, Birmingham, UK

**Keywords:** Psychology, Human behaviour

## Abstract

This study focuses on the cross-cultural adaptation of the Emotional Outburst Questionnaire (EOQ) to Brazilian Portuguese and preliminarily assesses its predictive validity. The EOQ evaluates aspects of emotional outbursts (EO), including frequency, duration, intensity, types, associated behaviours, recovery time, triggers, and effectiveness of calming strategies. Two independent translators performed the translation, with subsequent synthesis and analysis revealing that only 33 items (24.81%) required revision. Among these, one item needed partial modification, and two needed total modification. The study demonstrated strong content validity and adaptation in terms of conceptual, idiomatic, and semantic aspects. The EOQ's predictive validity was assessed by analysing the interruption of mental health services in Brazil due to Covid-19 (T1) compared to when services resumed after social distancing measures were lifted (T2). Parents of 25 individuals with developmental disabilities (ASD, DS and ID), with a mean of 11 y/o, mostly male (76%), completed the EOQ. Service interruption during T1 led to increased frequency and duration of severe emotional outbursts reported by caregivers compared to T2 (frequency: *p* < .001; duration: *p* = 0.05). This suggests that the EOQ exhibits predictive validity and sensitivity to changes influenced by individual contexts. These findings highlight the EOQ's potential as an outcome measure for intervention development.

## Introduction

Emotional outbursts (EO) are dysfunctional behaviors highly prevalent in children and adolescents with severe neurodevelopmental disabilities such as Autism Spectrum Disorder (ASD) and intellectual disability (ID) that can require lifelong care interventions^1^ as they significantly impact adaptive functioning^2,3^. Previous studies use other related terms for emotional outbursts such as 'meltdowns', 'crisis', 'behavioural breakdown', 'blips', 'rages', 'temper outbursts', 'tantrums', or 'tempers'^3–5^. For standardization purposes, the term emotional outburst (EO) will be adopted in this article. The types of EO commonly reported in studies include behaviours such as crying, screaming, going limp, flailing, hitting, throwing items, breath-holding, pushing, biting, destroying property or injuring other people or themselves, among others^3^.

In typical development, the frequency of EO is expected to decrease throughout childhood, mainly because of the acquisition of cognitive, communication and socio-emotional skills that provide an emotional and behavioral repertoire with better adaptive function according to the demands of the environment^2,6^. Previous studies reported that the highest rates of EO in typical development occur in children aged 1 to 4 years old, with an approximate frequency of 1 to 5 times a day and a duration of up to 15 min^7,8^. This frequency tends to decrease with increasing age. However, when the emotional outbursts persist after the age of four and are accompanied by aggressive behaviours or self-harm, it is recommended to assess expected developmental milestones or to screen for signs of neurodevelopmental or other psychiatric conditions^2^.

Emotional dysregulation is characterized by too much emotion, expressed too often, too quickly, and associated with antecedent triggering events^9^. Negative emotional dysregulation is usually the main feature characteristic of emotional outbursts. Children with severe neurodevelopmental disabilities such as ID and ASD have high rates of EO, even after the age of five^2,3,10,11^. In autism, the deficits in emotional dysregulation affect more than 80% of individuals with the diagnosis, which may be linked to specific patterns of functioning and connectivity in the amygdala/prefrontal cortex as well as difficulties in the processing of social information^12^. These factors may influence emotional dysregulation increased EO and impaired adaptive functioning^13–15^.

The assessment of emotional outbursts can be done using inventories, questionnaires, scales, behavioural observation and structured and semi-structured interviews^16^. One of the advantages of using inventories, questionnaires and scales is the systematization of measurements in all parameters (for example, type or topography, frequency, duration, among others). Supporting this view, research identifying characteristics of the development of emotional self-regulation in individuals under the age of 18 years, mainly in natural contexts suggested that it is necessary for researchers to consider the richness involved in observing behavioural parameters such as duration and sequence of behaviours^17^.

Several instruments assess emotional dysregulation through behavioural parameters^9^. The Emotion Dysregulation Inventory (EDI) evaluates emotional dysregulation within the past seven days using a Likert scale, covering emotional reactivity and dysphoria^18^. The Emotion Regulation Checklist (ERC) measures emotional lability/negativity and emotion regulation through Likert scale responses^19^. The Emotional Outburst Inventory (EMO-I) screens phasic irritability/severe emotional outbursts in clinical youth settings, evaluating outburst severity, frequency, and duration^20^. Additionally, the Preschool Age Psychiatric Assessment (PAPA)^21^ and Child and Adolescent Psychiatric Assessment (CAPA)^22^, both available through Duke University, gather specific information on tantrum severity, independent of mood. CAPA is designed for children aged 9 to 17, while PAPA is for children aged 2 to 8.

Another instrument is the Emotional Outburst Questionnaire (EOQ), which was developed to fill gaps in the literature regarding the assessment of emotional outbursts and their associated contexts and mechanisms. The EOQ comprises 133 items that assess different behavioural indicators of EO such as their frequency, duration, emotional patterns during their occurrence, recovery time, physiological and environmental factors that trigger EO and the effectiveness of strategies used by parents, caregivers and professionals to reduce EO^23^.

In low and middle-income countries^2,23^, there are no instruments with appropriate psychometric properties tested for validity that can comprehensively assess EO indicators. In Brazil, for instance, there are only two instruments that can be used to assess severe behavioural problems in individuals with neurodevelopmental disabilities such as ASD and ID. These include the Behavior Problems Inventory (BPI-01)^13,24^ and the Aberrant Behavior Checklist (ABC)^25^. Both are designed to assess certain types of severe behavioural problems but are not specific instruments for the evaluation of different parameters of EO as they are measured in the EOQ.

Given the negative impacts that EO can have, both for the person who experiences them and for parents, caregivers and other professionals, there is a need for culturally adapted instruments to evaluate them that have evidence of validity in the cultural and social contexts in which they will be used. To assure the quality and reliability of a measurement tool, it is important that when adapting instruments for use in different cultures, a methodologically rigorous process is used^26–28^.

The objective of this study was to describe the translation and cross-cultural adaptation of the EOQ for use in the Brazilian context, and to verify predictive validity evidence based on external criteria of the instrument. We hypothesized that EO would increase during a period of interruption of mental health services due to the COVID-19 pandemic.

## Methods

This was a cross-sectional quantitative study to examine the content validity and validity based on external criteria of the Brazilian Portuguese version of the EOQ. The study sample was selected by convenience. In addition to translation and cultural adaptation of the instrument, feedback from members of the target population was adopted to increase content validity. For evidence of predictive validity, interruption of mental health services during the COVID-19 pandemic was used as an external criterion. We hypothesized that EO would increase due to a lack of access to mental health services. The project was approved by the Human Research Ethics Committee of the Mackenzie Presbyterian University (Process: CAAE-29428620.4.0000.0084) and all the caregivers provided informed consent prior to participating in this study and completing the Brazilian Portuguese version of the Emotional Outburst Questionnaire.

### Instrument

Emotional Outburst Questionnaire: the instrument assesses EO based on the reports of informants, with the recommended informants being parents or caregivers^23^. The average completion time for the sample of Brazilian caregivers is 1 h. The EOQ comprises 133 items grouped into six factors: (a) Types of EO: behaviours that occur during EO according to the level of severity, namely: aggression directed at others, property and oneself; vocalizations; motor agitation and avoidance, among others. The frequency of the behaviours during EO are coded as “does not apply/never/rarely” when they occur 0 to 3 times in every 10 outbursts; “sometimes” when they occur between 4 and 6 times in every 10 outbursts, and “often/always” when they occur between 7 and 10 times in every of 10 outbursts. The frequency of the occurrence of EO has the response options “never”, “less than once a month”, “once a month”, “two to three times a month”, “once a week”, “two to three times a week”, “once a day” and “more than once a day”; (b) EO duration: answered by means of a timeline, with options of "less than 5 min", "5 to 15 min" "15 to 30 min", "30 min to 1 h", "1 to 2 h", "2 h to 1 day" and "one day or more"; (c) Emotional patterns: evaluated through a scale scored from 1 to 7, with a variability between “not angry or upset at all” to “as angry or upset as I have ever seen them”; (d) EO recovery time: measures the time required to recover an EO, classified as: “less than 5 min”, “5 to 15 min”, “15 to 30 min”, “30 min to 1 h”, “1h to 2h”, “2h to 1 day” and “one day or more”; (e) Physiological and environmental factors that trigger EO: factors such as hunger, tiredness, pain, and stressful events such as changes in routine; (f) Control strategies used to calm the person during EO: evaluates the use of strategies such as persuasion, physical comfort, relaxation, use of visual aids, punishment, and negotiation, among others.

A previous study was conducted to assess whether the contextual clusters of emotional outbursts evaluated using the EOQ that emerged from a sample of caregivers in Brazil would be comparable to the clusters identified in a study from the United Kingdom^29^. To evaluate this cross-cultural comparison, the factor structure of the contextual items in the Brazilian Portuguese version of the questionnaire was validated and compared against the factors derived from the English version^29^. Additionally, the EOQ can be completed by different informants, making it possible to provide information on clinical aspects of the emotion regulation of individuals with neurodevelopmental disabilities based on multiple informants.

### Procedures and participants

The process of translation, cross-cultural adaptation, and content and predictive validity analysis was conducted in four stages. We adopted the recommendations of the International Test Commission in respect to the methodological procedures used for the cross-cultural adaptation of the instrument^28,30^:

#### Stage 1—translation and cross-cultural adaptation

Independent translations by two professionals: both translators are native speakers of Portuguese and are proficient in English (MCTV and RL), have master's and doctoral degrees and more than 30 years' experience in behavioural assessment and intervention, and neurodevelopmental disabilities. After the two translations were completed, they were synthesized by considering semantic, experiential, idiomatic and conceptual equivalence in respect to the items^27^. Semantic equivalence was determined by assessing whether the words had the same meaning and whether any item had more than one possible meaning, or whether there were grammatical errors in the translation. The determination of idiomatic equivalence assessed whether the items that were difficult to translate from the original instrument were adapted and translated using an equivalent expression that did not change the cultural meaning of the item. Experiential equivalence was determined by assessing whether a given item was appropriate in the new culture and, if not, it was replaced by an equivalent item. Finally, the determination of conceptual equivalence assessed whether a given term or expression, even when translated properly, evaluated the same aspect in different cultures.

#### Stage 2—back-translation and final modifications

The back-translation was carried out into the original language by a certified native English-speaking translator and a specialist in Portuguese-English translation. The author of the original version of the EOQ (JC) then evaluated the back-translation to verify that the content of the back-translated items was compatible with the content of the original items.

#### Stage 3—evaluation by target audience

Seven parents of children with ASD between 11 and 25 years old (mean = 18.28, *SD* = 5.34, sex male = 5), were selected by convenience to complete the EOQ to verify that they correctly understood the content of the items. This was based on the following questions: (a) Did you understand everything that was asked? (b) Could you explain what you understood with examples of behaviour of your child/student/adult in respect to each item? (c) Were there any specific words that you did not understand?

#### Stage 4—preliminary evidence of predictive validity

During the COVID-19 pandemic, around 70% of the individuals with ASD experience social isolated, when at least 30% of in-person treatments were completely interrupted in Latin America, including Brazil^31^. This atypical situation allowed us to test differences in the frequency and severity of EO during this period (Time 1) compared to when services were resumed in 2022 (with the improvement of the health situation in respect to COVID-19 - Time 2). Between June 2020 and January 2021, a sample of participants with developmental disabilities was selected by convenience. This sample of 25 parents of children and adolescents diagnosed with Down syndrome (DS), ID or ASD completed the EOQ (mean= 11 years old; *SD* = 4.89; range = 5–25 years old). Parents were informed about the study and invited to respond to the questionnaire through social media of parent support groups and during visits to mental health service clinics with their children. A total of 201 parents were invited to participate and all parents who participated in the present study accepted this invitation. This low response rate might have been due to reasons such as (a) change of email contact; (b) invitations being sent directly to spam. None of the parents who did not participate in the research responded by declining the invitation. Participants were invited in person (those who attended the clinic) or directly via email message (for those who had already completed the questionnaire directly via the RedCap platform link). For participants who were personally invited to the clinic and who agreed to participate, a tablet with the questionnaire was made available to fill out the questionnaire, while the participant waited for their child's intervention session. Participation in the study was voluntary and participants did not receive any financial reward for the study. However, a link to an online talk was provided to parents who completed the questionnaire. The theme of the talk was “Parental behavioural management of children with developmental disabilities”. Only one mother attended the talk.

The sample size was determined based on convenience and practical constraints, primarily due to low response rates among participants who were already facing challenging circumstances as primary caregivers of individuals with neurodevelopmental conditions. To minimize any additional burden on them, we refrained from insisting on their participation. Given these constraints, a formal power analysis was not conducted. The study's findings should be interpreted with caution, particularly in terms of generalizability. Further discussion on this aspect is provided in the Limitations section.

Time 1 of data collection corresponds to the worst period of the COVID-19 pandemic in Brazil, when mental health services were interrupted. Time 2 was in 2022 when Brazil adopted no restriction measures of social distancing. The caregivers completed the EOQ online on the Research Electronic Data Capture (REDCap) platform. After the improvement in the health situation in respect to COVID-19 and the resumption of face-to-face mental health interventions, between the months of May and August 2022, parents completed the EOQ again to evaluate whether the instrument could temporally predict outcomes in regards to the frequency and severity of EO using access to mental health services as an external independent variable.

### Data analysis

In the analysis of the translations for the synthesis process, the two professionals compared the two translations and analysed idiomatic, conceptual/experiential, and semantic discrepancies. For the analysis of predictive validity, the EO indicators evaluated by the EOQ were compared between the two time points using the non-parametric Wilcoxon signed-rank test, adopting a statistical significance level of *p* ≤ 0.05. The instrument indicators that were evaluated were those that assessed the frequency of outbursts (most severe, least severe, and general), duration of outbursts (most severe and least severe), and intensity of outbursts (most severe and least severe). For the analyses, the response options were classified numerically: for example, in items relating to the frequency of the outburst, “never” was coded as “0”, and “more than once a day” was coded as “7”. Additionally, we asked the caregivers if they believed that their child's behaviours had changed after the relaxation of COVID-19 prevention measures, followed by a question on whether they believed the changes were for better or worse.

### Ethical approval

The study procedures were carried out in accordance with the Code of Ethics of the World Medical Association (Declaration of Helsinki) and were approved by the Research Ethics Committee of the Mackenzie Presbyterian University (Process number: CAAE-29428620.4.0000.0084).

## Results

After carrying out the two translations, analyses were performed based on identifying idiomatic, conceptual/experiential, and semantic discrepancies for the synthesis composition. Out of a total of 133 items, 33 (24.81%) required revision (including the instructions). Table 1 shows the 33 items that required revision according to the criterion used (semantic, experiential/conceptual and/or idiomatic). One item required partial modification and two total modifications. There was good evidence of content validity and the adequacy of the adaptation in respect to the conceptual, idiomatic, and semantic aspects of the items of the instrument.

**Table 1:** Results of the translation synthesis, back-translation, and final cross-cultural adaptation of the 33 items revised.

Original instruction or item	Translation synthesis	Back-translation	Final cross-cultural adaptation	Criterion and type of modification (TM, PM, UN)
In this questionnaire, we want you to think about the most severe and least severe emotional outbursts within the past month that the individual you care for has displayed and the characteristics associated with each type of emotional outburst, such as behaviours, frequency, and duration. In terms of the severity of emotional outbursts, we are ref erring to how disruptive and negatively impactful they are to the person and/or those around them at the time of the emotional outburst	Neste questionário, nós queremos que você pense sobre as explosões emocionais mais graves e menos graves que durante o mês passado a pessoa da qual você é o cuidador apresentou e as características associadas com cada tipo de explosão emocional, tal como comportamentos, frequência e duração. Em relação à gravidade das explosões emocionais, nós estamos nos referindo ao grau de disrupção e ao impacto negativo que essas explosões têm para a pessoa e/ou àqueles ao redor dela no momento da explosão emocional	In this questionnaire, we would like you to think about the more and less serious emotional outbursts that the person f or whom you are the caregiver has displayed over the past month and the characteristics associated with each type of emotional outburst, such as behaviours, frequency, and duration. With regards to the seriousness of the emotional outbursts, we are ref erring to the level of disruption and to the negative impact that these outbursts have for the person and/or for those around them at the time of the emotional outburst	Neste questionário, nós queremos que você pense sobre as explosões emocionais mais graves e menos graves que durante o mês passado a pessoa da qual você é o cuidador apresentou e as características associadas com cada tipo de explosão emocional, tal como comportamentos, f requência e duração. Em relação à gravidade das explosões emocionais, nós estamos nos referindo ao grau de disrupção e ao impacto negativo que essas explosões têm para a pessoa e/ou àqueles ao redor dela no momento da explosão emocional	Idiomatic (UN)
Meltdowns	Colapso emocional	Emotional breakdown	Crise	Idiomatic, conceptual and semantic (TM)
Blips	Crise temporária	Temporary crisis	Crise transitória	Idiomatic and conceptual (PM)
Rages	Explosões de raiva	Outbursts of anger’	Explosões de raiva	Idiomatic and conceptual (UN)
Tempers	Mudanças de humor	Mood swings	Mudanças de humor	Idiomatic, conceptual and semantic (UN)
Name-calling	Xingamentos	Swearing	Xingamentos	Idiomatic and semantic (UN)
Screaming	Berros	Shouting	Berros	Idiomatic and semantic (UN)
Shouting	Gritos	Screaming	Gritos	Idiomatic and semantic (UN)
Swearing	Falar palavrões	Using bad language	F alar palavrões	Idiomatic and semantic (UN)
Slamming door	Bater porta	Knocking on the door,	Bater porta	Idiomatic (UN)
Throwing objects down	Jogar objetos com força	Throwing objects with force	Jogar objetos com força	Idiomatic (UN)
Smashing windows	Estilhaçar vidros	Smashing glass	Estilhaçar vidros	Idiomatic (UN)
Grabbing	Agarrar	Gripping,	Agarrar	Idiomatic (UN)
Picking skin	Cutucar a pele	Poking their skin	Cutucar a pele	Idiomatic (UN)
Picking rectum	Cutucar o reto	Poking their rectum	Cutucar o ânus	Idiomatic and experiential (UN)
Pacing	Andar sem parar	Walking without stopping	Andar sem parar	Idiomatic (UN)
Rushing about	Correr	Running	Correr	Idiomatic (UN)
Increased physiological arousal	Resposta fisiológica aumentada	Increased physiological responses	Resposta fisiológica aumentada	Idiomatic (UN)
Contextually inappropriate sexual behaviours	Comportamentos sexuais em locais inapropriados	Sexual behaviour in inappropriate locations	Comportamentos sexuais em locais inapropriados	Idiomatic (UN)
Grabbing	Agarrar	Snatching	Agarrar	Idiomatic (UN)
Making themselves sick	Induzir vômitos	Inducing vomiting	Induzir vômitos	Idiomatic (UN)
Retching	Regurgitação	Regurgitation	Regurgitação	Idiomatic (UN)
Angry or upset	Nervosa ou emburrada	Nervous or grumpy	Nervosa ou emburrada	Idiomatic and experiential (UN)
As angry or upset as I have ever seen them	Nada nervosa ou emburrada	More nervous or grumpy than I’ve ever seen before	Nada nervosa ou emburrada	Idiomatic and experiential (UN)
Whilst on holiday away from home	Durante as férias longe de casa	During holidays far from home	Durante as férias longe de casa	Idiomatic (UM)
A parent/caregiver	Um parente/cuidador	A relative/carer	Um parente/cuidador	Idiomatic (UN)
Disagreement with others	Desentendimentos com os outros	Misunderstandings with others	Desentendimentos com os outros	Idiomatic (UN)
Being told off, criticised, or accused of making a mistake	Ser chamado a atenção, icado, ou acusado de fazer alguma coisa errada	Having attention drawn, criticized, or accused of doing something wrong	Ser chamado a atenção, criticado, ou acusado de fazer alguma coisa errada	Idiomatic (UN)
Being teased	Ser provocado	Being provoked	Ser provocado	Idiomatic (UN)
Someone not understanding the individual you care for	Alguém de f ora que não entende a pessoa que você cuida	Someone uninvolved doesn’t understand the person you care for	Alguém de fora que não entende a pessoa que você cuida	Idiomatic (UN)
Light is too bright	Iluminação fica muito clara	Lighting is very bright	Iluminação fica muito clara	Idiomatic (UN)
Temperature is too hot or too cold	Temperatura está muito quente ou muito fria	Temperature is very hot or very cold	Temperatura está muito quente ou muito fria	Idiomatic (UN)
Appearing withdrawn	Mostrar-se introvertido	Acting introverted	Retrai-se	Idiomatic and semantic (TM)
Staying in a bad mood	Ficar de mau humor	Getting in a bad mood	Ficar de mau humor	Idiomatic (UN)

PM, partially modified; TM, totally modified; UN, unmodified.

After the synthesis analysis, an analysis of the items was performed by seven mothers of children, adolescents, and adults with ASD (stage 3). None of the mothers reported any difficulties in understanding the items, instructions, and the response scale of the instrument, and completing the EOQ in approximately 1 h.

### Predictive validity of the EOQ

To explore the predictive validity of the EOQ using an external criterion, we conducted an analysis of a two-time point evaluation comparison based on an external independent variable—with the COVID-19 pandemic restriction measures (Time 1) and without the restriction measures (Time 2). The sample of this analyses consisted of 25 respondents of children, adolescents, and young adults, most of the children being male (76%), with 72% of the sample diagnosed with ASD, followed by 24% with DS and one child with an exclusive diagnosis of ID. The mean age of the and use of medication is reported in Table 2.

**Table 2:** Sample demographics among the evaluations in Time 1 and Time 2.

	Time 1	Time 2
Children's mean age (years old)	10.8 (*SD* = 4.89)	13 (*SD* = 5.02)
Medication use	44%	56%

The purpose of the medications was predominantly for behaviour management at both times, followed by attention stimulants, reported only at Time 2.

**Figure 1:**
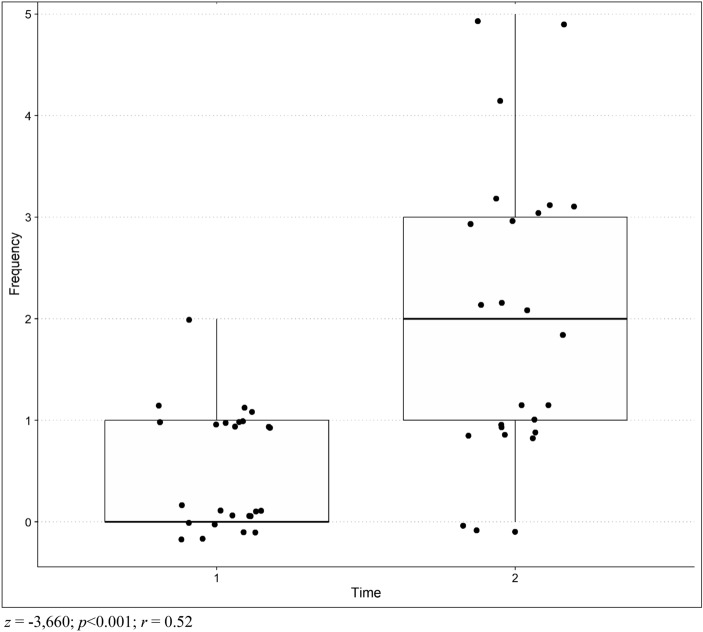
Difference between Time 1 and Time 2 comparing the frequency of the more severe emotional outbursts.

The Wilcoxon test showed that the frequency of more severe emotional outbursts increased over time, being higher at Time 2 than at Time 1 (*Z* = − 3.660; *p*<0.001), as shown in figure 1, with a relatively strong negative effect size (*r*=− 0.73). The same effect was not observed in questions referring to less severe (*Z* = − 1.615; *p* = 0.10) and general (Z = − 1.341; *p* = 0.18) emotional outbursts, as shown in Table 3.

**Table 3:** Difference between the frequencies of the most severe, less severe and general emotional outbursts in times 1 and 2 for frequency, duration and intensity questions (*n* = 25).

	Item	Time 1		Time 2		Difference between Times
		Minimum score	Maximum score	Minimum score	Maximum score	
Frequency	**Frequency of the most severe emotional outbursts (Question 24)**	**0**	**2**	**0**	**5**	***Z* =− 3.660** *p* < 0.001* ***r* = 0.73**
	Frequency of the least severe emotional outbursts (Question 52)	0	7	0	7	*Z* =**− **1.615 *p* = 0.10
	Frequency of general emotional outbursts (Question 57)	0	7	0	7	*Z* = **− **1.341 *p* = 0.18
Duration	**Duration of the most severe emotional outbursts (Question 25)**	**1**	**3**	**1**	**4**	***Z* = − 1.928** *p* = 0.05* ***r* = 0.38**
	Duration of the least severe emotional outbursts (Question 53)	1	4	1	3	*Z* = − 0.215 *p* = 0.83
Intensity	Intensity of the most severe emotional outbursts (Question 26)	1	7	1	7	*Z* = − 0.430 *p* = 0.66
	Intensity of the least severe emotional outbursts (Question 54)	1	5	1	7	*Z* = − 0.057 *p* = 0.95

The scored number are explained at the “Data analysis” section. * = *p* ≤ 0.05. Significant for 95% confidence.

Significant are in value [bold].

The item referring to the frequency of more severe emotional outbursts averaged 0.52, but increased to 1.96, on a scale from 0 (never) to 7 (more than one time a day), with 8 children whose number of outbursts did not change between the two times (32%).

**Figure 2:**
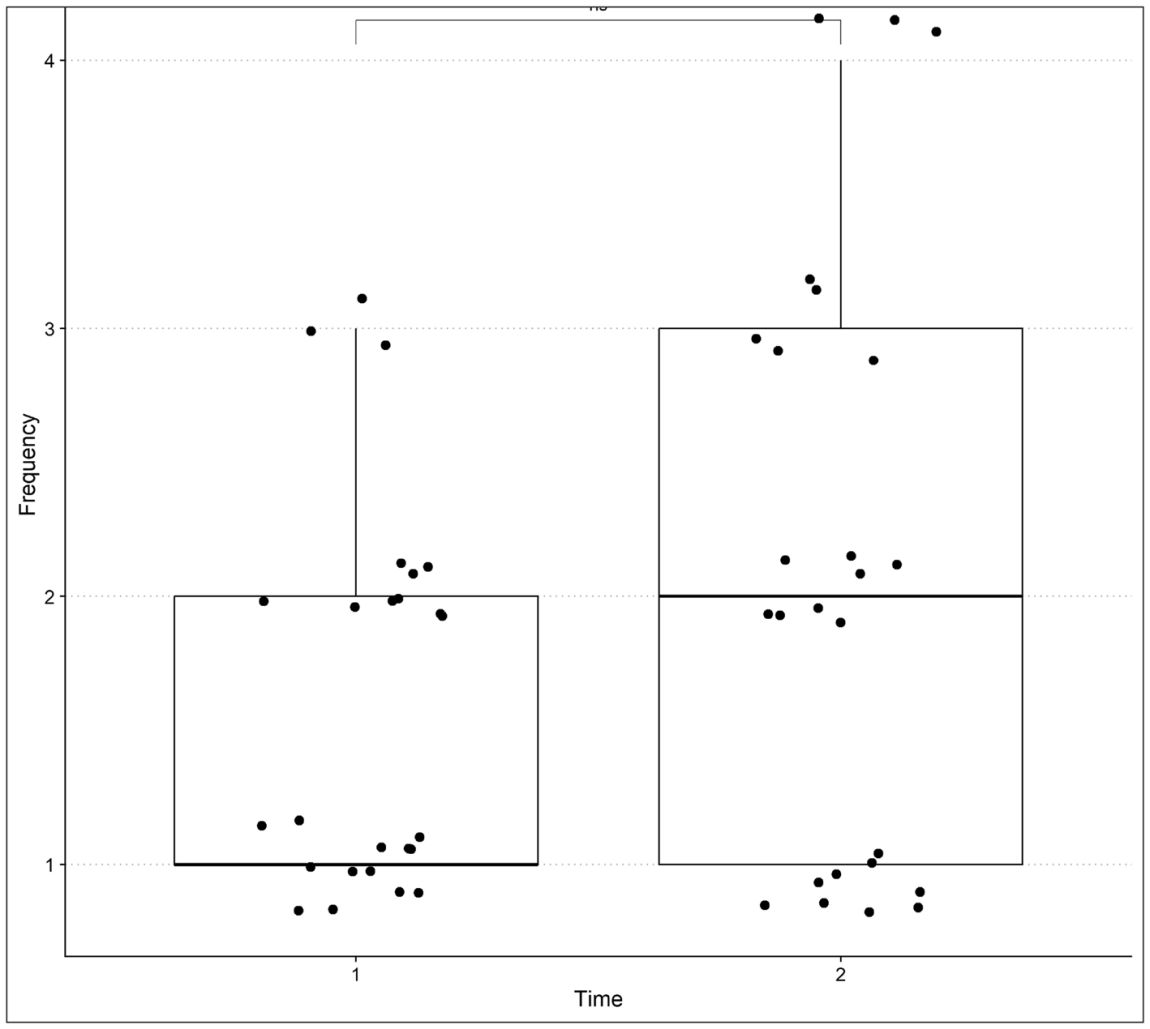
Difference between Time 1 and Time 2 comparing the duration of the more severe emotional outbursts.

Regarding the duration of the most severe emotional outbursts, there was a borderline increase in the time reported by caregivers (*p* = 0.05), with a moderate negative effect size (*r* = 0.38). At Time 1, parents (52%) reported that the outbursts lasted less than 5 min, with a maximum of 15 to 30 min in 3 cases (12%). At Time 2, three parents (12%) reported duration of outbursts from 30 min to 1 h, which was not observed in the first assessment, although most responses (36%) indicated outbursts of less than 5 min at Time 2 (Fig. 2).

At both times, the use of mental health services was evaluated, with the types of care provided by professionals in psychology, speech and language therapy, occupational therapy, neurologists, psychiatrists, and others. At time 1 (during the worse period of the Pandemic COVID-19 lockdown) 72% (*n* = 18) of the children in the sample received mental health services, while at time 2 this number increased to 92% (*n* = 23).

There was a significant increase in the frequency and duration of emotional outbursts in the present study sample at the Time 2. It is possible that this increase is associated with lack of access to mental health services in approximately 30% of the sample at Time 1.

At Time 2, parents were asked about their general perception of their children's behaviours, with the question: “Do you believe that your child's behaviours have changed after the relaxation of COVID-19 prevention measures?”, followed by the question: “If yes, do you believe that the changes were for better or worse?”. As a result, 60% of parents reported observing changes in their children's behaviour from Time 1 to Time 2, and 52% reported that the changes were for better (six parents did not answer this question in the Time 2 evaluation).

## Discussion

The present study described the process of translation and cross-cultural adaptation of the EOQ for use in Brazil, and preliminary evidence with regards to its content and predictive validity^23^. The EOQ assesses the frequency and duration of emotional outbursts (EO), types of EO, emotional patterns during the EO, recovery time from EO, environmental and physiological factors that trigger EO and the effectiveness of control strategies used to calm individuals with EO. It is important that EO assessment tools are available to monitor indicators of emotional dysregulation in people with severe neurodevelopmental disabilities. It is important to have culturally adapted instruments for specific contexts because many autism studies tend to overlook the complex interplay of several factors affecting this population and how professionals approach the diagnosis and intervention practices. Conducting research that is culturally, gender, racial, and ethnically sensitive could result in more accurate and valid procedures for a diverse range of autistic individuals^32^.

The process of translation, cultural adaptation and synthesis showed that the Portuguese version was adequate since there were few errors in relation to the presence of complex and elaborate words or phrases. We verified that only minor adjustments to semantic, experiential/conceptual and/or idiomatic aspects were required. Despite the large number of items of the EOQ, only 24.81% of items/instructions required modification. This result demonstrates the quality of the translations and provides support for the content validity of the questionnaire^27,28^. As we hypothesized, the Brazilian Portuguese version of the EOQ maintained its measurement properties and is equivalent to the original version, indicating a successful adaptation. This process made the EOQ suitable for use in the research area in Brazil.

For predictive validity, we used the interruption of mental health services during the COVID-19 pandemic as an external criterion. Our hypothesis was that EO would increase due to the lack of access to mental health services, and as expected, differences emerged between Time 1 and Time 2. This provides evidence of the EOQ's sensitivity in measuring changes influenced by an individual's environmental context. Specifically, at Time 2, we observed a significant increase in the frequency and duration of emotional outbursts. Approximately 30% of the present sample did not receive intervention for nearly two years, possibly leading to more pronounced impacts and heightened EO. An international study^33^ also observed increased intensity of behaviour problems in individuals with ID during lockdown, potentially due to reduced stimulation. Additionally, evidence also suggests that the interruption of mental health services had detrimental effects on ASD populations, with families noting behavioural setbacks during confinement^31^. Even with interventions resuming, EO levels had not fully returned to pre-pandemic levels, indicating no significant improvement in behaviour from the parents' perspective in our study. This corroborates recent literature, which indicates that challenging behaviours, such as emotional outbursts, are chronic in the clinical conditions of individuals with ID or ASD^34^.

The indicators of frequency and intensity of emotional outbursts worsened or remained the same from Time 1 to Time 2, except for the duration of less severe emotional outbursts, which decreased by 1 point in the maximum score. This result does not compromise the predictive validity of the instrument, and it can be hypothesized that for this reduction in scores, there is evidence from previous studies showing that removing demands, especially social ones such as attending school^35^, was a driver of well-being for autistic pupils and their parents/caregivers^36^.

The increased use of medication from Time 1 to Time 2 corroborates the data found by Rauf et al.^37^, who also reported an increase of medication use in a similar sample during the pandemic. In an attempt to support people with ID and their families in managing behavioural problems, a rise in requests for psychotropic medication was expected^38^. Since the medications were mostly for behavioural purposes for Rauf et al.^37^ as well as our study, their use could have been for EO, which also increased from Time 1 to Time 2. Although medication use is not a direct question in the (EOQ), it is a variable to be considered when assessing behavioural problems and emotional outbursts, especially if the medication purpose is for the management of behavioural problems.

Given the scarcity of scientific evidence on the contexts and mechanisms associated with emotional outbursts^23^, the present study contributes to not only the verification of preliminary predictive validity of the Brazilian version of EOQ, but also the understanding of the influence of the environment on the emotional outbursts of neurodivergent individuals, such as ASD and ID populations. Our research paves the way for significant contributions in the realms of potential intervention and recommendations for practice, education, and management. It is essential to understand the factors contributing to emotional outbursts, particularly in populations with ASD and ID diagnoses^1,34,39^. This understanding is a fundamental step in designing and monitoring the effectiveness of targeted interventions, which may encompass behaviour management strategies, stress reduction techniques, and support systems for caregivers, educators, and healthcare professionals. Future research should focus on the design and evaluation of interventions that can mitigate emotional dysregulation in these populations.

This study demonstrates that the Brazilian version of the EOQ is promising, but also has some limitations. The study's findings should be interpreted with caution, especially regarding their generalizability, as the sample size and heterogeneity in the age of the participants may limit the ability to detect smaller or more nuanced effects and can limit the interpretation of the data. Future studies may benefit from conducting a power analysis to determine an appropriate sample size, and we emphasize the importance of including larger and more representative samples in research efforts. While the sample size may not be ideal, it is worth noting that our research still provides valuable insights and contributes to the existing knowledge. In terms of future directions, further studies are recommended with the expansion of the sample and inclusion of new variables, such as interrater reliability, exposure to screens and relaxation of parental rules/exercise of parenting. It would also be interesting for future studies to replicate the EOQ in developing countries with similar cultural contexts, such as Latin America, as done by Chung et al.^23^ in developed countries.

Another point to be studied is the association between the frequency and severity of emotional outbursts and the frequency and type of mental health services. Further research on the predictive validity of the EOQ could involve other criterion variables, such as estimating the predictive validity of the EOQ with other populations, especially ID, to verify its association with the level of impairment (e.g., repetitive behaviours, language, cognitive, function, language, and motor impairments). In addition, new studies on concurrent validity using other criteria should be conducted. It is also important to note that not all EOQ variables evidenced worsening in severity, and this relates to a limit of the measure, which does not comprise an overall severity score.

In the last question, after Time 2 (general perception of change in their children's behaviours), it was possible to note that about 40% of the parents reported no change. However, the main results, based on the EOQ questions, have shown a significant increase reported by caregivers. This difference could be linked to memory biases or maybe a more general and less specific view of behaviours, because parents were asked about general changes and not specific behaviours or their severity, frequency or intensity.

## Conclusion

The present study described the process of translation and cross-cultural adaptation of the EOQ for use in Brazil, and preliminary predictive validity. It is important that EO assessment tools are available to monitor indicators of emotional dysregulation in people with severe neurodevelopmental disabilities. It is recommended to have studies of psychometric properties of instruments that were translated from other countries when considering the use of an instrument. The analysis of external validity revealed, even if preliminarily, that the EOQ is sensitive in measuring changes influenced by the individual's environmental context. However, even with these results on validity, the verification of predictive validity of the EOQ still requires further studies to explore the influence of other environmental and cultural factors as well as the assessment of other severe neurodevelopmental disabilities, e.g., intellectual disability. Other psychometric studies are required to verify the accuracy of validity and reliability of the EOQ in the Brazilian context. Nevertheless, this study brings not only psychometric evidence for the EOQ, but also contributes to the understanding of the influence of the environment on the emotional outbursts of neurodivergent individuals. In practice, the results of our study emphasize the importance of culturally adapted assessment tools for emotional outbursts and the understanding of environmental or biological factors that may contribute to emotional outbursts in neurodivergent individuals.

## Data Availability

The data analysed in this study can be found at https://osf.io/qtser/.
